# Supplementary material for: Patterns of Intron Gain and Loss in Fungi
Source: PLoS Biol. 2004 Nov 30;2(12):e422. doi: 10.1371/journal.pbio.0020422 (PMC532390; doi:10.1371/journal.pbio.0020422)
Supplement: Table S1 — Also available at http://genes.mit.edu/NielsenEtAl/. (4.3 MB ZIP). [file pbio.0020422.st001.zip › NielsenEtAl/html/594.html]

AN0081.1.NCU00440.1.MG05201.1.FG04104.1


```
 CLUSTAL W (1.82) Multiple Sequence Alignments - Introns Inserted


Sequence 1: MG05201.1	359 aa
Sequence 2: FG04104.1	329 aa
Sequence 3: NCU00440.1	328 aa
Sequence 4: AN0081.1	352 aa
Alignment Length: 359 aa
Number Identitical Residues: 258 aa
Alignment Score (without introns) 11139


MG05201.1 	MDSQRSNEVSPEAMQARIQQARREAETLKDRIKRKKDELADTTL1RTVAQQAHEPIPKNQ
NCU00440.1	MDSR-SSDVSPEAMQARIQQARREAENLKDRIRLKKEGLADTSL1YEVAQQAHEPLPKNN
FG04104.1 	MNSQGNNDVSPEAMQSRIQQARREAETLKDRIKRKKDDLADTTL1RAVAQQAHEPIPKNQ
AN0081.1  	-----MADMSGEQMQAKITAARREAEGLKDKIRRRKDDLADTTL1RDVAQNQTDALPR-I
          	       ::* * **::*  ****** ***:*: :*: ****:*   ***:  :.:*:  

MG05201.1 	LMKAKRTLKGHLAKIYAMHWSTDRRHLVSASQDGKLIIWDAYTTNKVHAIPLRSSWVMTC
NCU00440.1	MMKTKKTLKGHLAKIYAMHWSTDRRHLVSASQDGKLIIWDAYTTNKVHAIPLRSSWVMTC
FG04104.1 	LMKAKRTLKGHLAKIYAMHWSTDRRHLVSASQDGKLIIWDAYTTNKVHAIPLRSSWVMTC
AN0081.1  	GMKPRRTLKGHLAKIYAMHWSTDRRHLVSASQDGKLIIWDAYTTNKVHAIPLRSSWVMTC
          	 **.::******************************************************

MG05201.1 	AYAPSGNYVACGGLDNICSIYNLNQNRDGPTRVARELSGHAGYLSCCRFINDRSILTSSG
NCU00440.1	AYAPSGNFVACGGLDNICSIYNLNSNRDGPTRVYRELSGHAGYLSCCRFINDRSILTSSG
FG04104.1 	AYAPSGNFVACGGLDNICSIYNLNQQRDGPTRVARELSGHAGYLSCCRFINDRSILTSSG
AN0081.1  	AYAPSGNYVACGGLDNICSIYNLSS-REGPTRVARELSGHSGYLSCCRFINDRRIITSSG
          	*******:***************.. *:***** ******:************ *:****

MG05201.1 	DMTCMKWDIETGTKVIEFADHLGDVMSISLNPTNQNTFISGACDSFAKLWDIRAGKAVQT
NCU00440.1	DMTCMKWDIETGTKVVEFADHLGDVMSISLNPTNQNTFVSGACDSFAKLWDIRAGKAVQT
FG04104.1 	DMTCMKWDIETGQKVTEFADHLGDVMSISLNPTNQNTFISGACDAFAKLWDIRAGKAVQT
AN0081.1  	DMTCMLWDIESGSKVTEFADHLGDVMSISINPTNQNIFVSGACDAFAKLWDIRTGKAVQT
          	***** ****:* ** *************:****** *:*****:********:******

MG05201.1 	FAGHESDINAIQFFPDGHSFVTGSDDATCRLFDIRADRELNVYG0SESILCGITSVATSV
NCU00440.1	FAGHESDINAIQFFPDGHSFVTGSDDATCRLFDIRADRELNCYR0---------------
FG04104.1 	FAGHESDINAIQFFPDGHSFVTGSDDATCRLFDIRADRELNLYG0---------------
AN0081.1  	FAGHESDINAIQFFPDGNAFGTGSDDTTCRLFDIRADRSLNTYQ0SDQILCGITSVGFSV
          	*****************::* *****:***********.** *  :..  .. :: . : 

MG05201.1 	SGRLLFAGYDDFECK0VWDITRGDKVGSLVGHENRVSCLGVSNDGISLCTGSWDSL0LKI
NCU00440.1	---------------~VWDLTRAEKVGSLVGHENRVSCLGVSNDGISLCTGSWDSL0LKV
FG04104.1 	---------------~VWDITRGEKVGSLVGHENRVSCLGVSNDGTSLCTGSWDSL0LKI
AN0081.1  	SGRLLFAGYDDFECK0VWDVLRGDKVGSLSGHENRVSCLGVSNDGISLCTGSWDSL0LKV
          	:.    :. .. ... ***: *.:***** *************** ********** **:

MG05201.1 	WAY
NCU00440.1	WAY
FG04104.1 	WAY
AN0081.1  	WAW
          	**:
```
